# Partners in the Warburg effect

**DOI:** 10.7554/eLife.15938

**Published:** 2016-04-13

**Authors:** Joshua D Rabinowitz, Hilary A Coller

**Affiliations:** 1 Department of Chemistry and the Lewis Sigler Institute for Integrative Genomics, Princeton University, Princeton, United Statesjoshr@princeton.edu; 2 Department of Molecular, Cell and Developmental Biology, University of California, Los Angeles, Los Angeles, United States; 3 Department of Biological Chemistry, David Geffen School of Medicine, Los Angeles, United States

**Keywords:** cancer metabolism, tumor microenvironment, exosomes, metabolic flux analysis, reductive carboxylation, macropinocytosis, Human

## Abstract

Cells that surround tumors produce vesicles that supply nutrients to cancer cells and, more surprisingly, also impair the generation of energy in these cancer cells.

**Related research article** Zhao H, Yang L, Baddour J, Achreja A, Bernard V, Moss T, Marini JC, Tudawe T, Seviour EG, San Lucas FA, Alvarez H, Gupta S, Maiti SN, Cooper L, Peehl D, Ram PT, Maitra A, Nagrath D. 2016. Tumor microenvironment derived exosomes pleiotropically modulate cancer cell metabolism. *eLife* **5**:e10250. doi: 10.7554/eLife.10250

**Image** Cancer cells (pale blue) receive vesicles (dark blue) from neighboring cells (yellow)

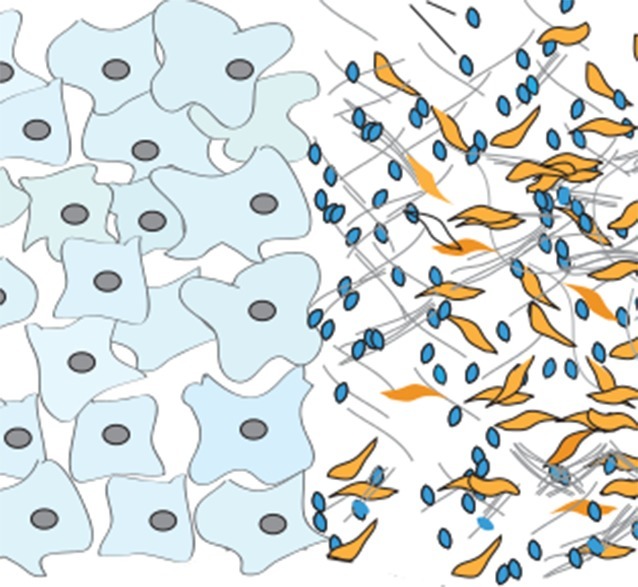

In 1918 Albert Einstein convinced Otto Warburg to leave the German infantry and fulfill his patriotic requirements in the first World War by performing research instead (Koppenol et al., 2011). Back in the lab at the Kaiser Wilhelm Institute, Warburg discovered that thin slices of tumors produced lactate much more rapidly than normal tissue. This rapid fermentation of glucose by tumors, even in the presence of ample oxygen, was the first biochemical trait assigned to cancer and is known as the Warburg effect.

When oxygen is present, most human cells rely on a process called oxidative phosphorylation inside mitochondria to convert lactate into carbon dioxide and usable energy. Warburg proposed that the rapid glucose fermentation and associated lactate secretion by the cancer cells was due to mitochondrial dysfunction. However, subsequent studies have shown that most cancer cells do have working mitochondria and, moreover, depend heavily upon them to produce energy (Zu and Guppy, 2004; Moreno-Sánchez et al., 2007). Instead of causing mitochondrial dysfunction, it was found that the mutations that cause cancer also promote the breakdown of glucose in a process called glycolysis. The most striking example involves the PI3K-Akt signaling pathway, which both transduces the signal from the hormone insulin to drive glucose uptake, and is one of the most frequently mutated pathways in cancer. One way this pathway can be activated is by the loss of a tumor suppressing enzyme called PTEN (Shaw and Cantley, 2006). The observation of oncogene-driven glucose uptake seemed to neatly explain the Warburg effect.

Over the past few decades, evidence has steadily accumulated that cancer cells also hijack surrounding cells (Cirri and Chiarugi, 2012). For example, cancer cells secrete growth factors to promote the formation of new blood vessels (Orimo et al., 2005), which are required to supply tumors with nutrients. Moreover, they co-opt surrounding connective tissue cells, including fibroblasts, which exchange signals with the cancer cells in a manner that ultimately drives tumor growth and likely helps to suppress immune responses to the tumor (Cirri and Chiarugi, 2012). However, both the mechanism of this exchange and its role in tumor growth remain poorly understood.

Fibroblasts may exchange both signaling molecules and metabolic fuels with the cancer cells, either by secreting individual molecules (e.g. lactate; Martinez-Outschoorn et al., 2014) or by releasing membrane-bound vesicles known as exosomes (Castellana et al., 2009). For example, recent work has shown that the spread of cancer in the brain is promoted by the exosomes that are released by a particular type of brain cell. These exosomes contain small RNA molecules known as microRNAs that can silence the gene that encodes the PTEN enzyme, whose loss drives an increase in glycolysis (Zhang et al., 2015).

Now, in eLife, Deepak Nagrath at Rice University and colleagues – including Hongyun Zhao as first author – show that cancer-associated fibroblasts release exosomes that both deliver nutrients to cancer cells and inhibit oxidative phosphorylation (Zhao et al., 2016; Figure 1). Zhao et al. use isotope-labelled carbon compounds to provide compelling evidence that exosomes from fibroblasts can supply an amino acid called glutamine and other nutrients to cancer cells. A shortage of glutamine can limit the growth of pancreatic and perhaps other cancers (Kamphorst et al., 2015). Importantly, although the exosomes contribute modest amounts of nutrients, they can protect cancer cells from starvation, hinting at one potential role for such metabolic exchange in tumors.

More striking and surprising is the role of the exosomes in causing the Warburg effect. Adding exosomes to prostate or pancreatic cancer cells both promotes glycolysis and blocks oxidative metabolism. It is likely that the increase in glycolysis is caused by the reduction in oxidative phosphorylation so, in this respect, the exosomes trigger glycolysis in the way initially envisioned by Warburg. These results call for a re-examination of the contributions of both processes to energy generation in cancer cells that are still associated with their neighbors.

Such re-examination is particularly important given that oxidative phosphorylation is reduced so dramatically in cancer cells, with oxygen consumption lowered by up to 80% within 24 hours of receiving exosomes from fibroblasts. Zhao et al. – who are based at Rice University, Baylor College of Medicine, the University of Texas MD Anderson Cancer Center and Stanford University – propose that the exosomes may deliver microRNAs that silence oxidative metabolism genes, but this is hard to reconcile with the timing. Since the proteins involved in oxidative phosphorylation are generally long-lived, even complete inhibition of their production seems unlikely to produce such drastic effects so quickly. Nor can the decreased oxidative phosphorylation be explained by the delivery of nutrients by exosomes, because increasing the access to such nutrients would be expected to promote, not inhibit, the use of oxygen. Thus, understanding how the exosomes inhibit oxidative phosphorylation is a key challenge going forward. Such work holds the potential to illuminate not only the Warburg effect, but also the regulation of oxidative phosphorylation in cells more generally.

**Figure 1.**
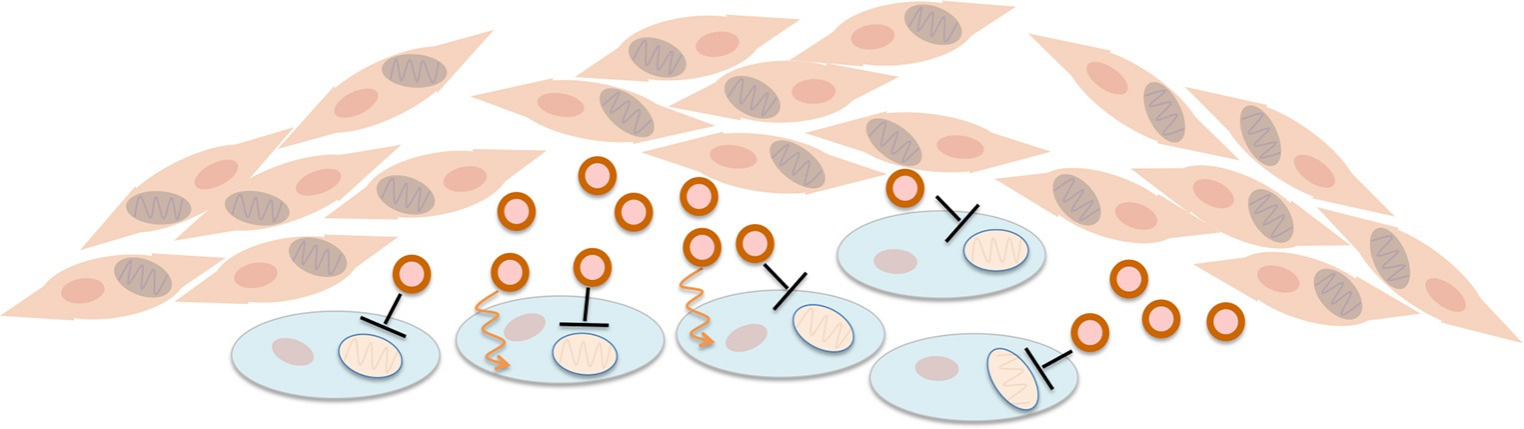
Fibroblasts supply nutrients to cancer cells and inhibit oxidative phosphorylation in cancer cells. Fibroblasts (pink cells) associate with epithelial cancer cells (blue cells) and release exosomes (circles) that transfer nutrients to epithelial cancer cells (orange lines). In addition, they inhibit mitochondrial oxidative phosphorylation in the cancer cells (black blunt arrows), perhaps via microRNAs that silence particular genes.
